# Real-world study of first-line therapy with aumolertinib for elderly patients with non-small cell lung cancer harboring EGFR mutation

**DOI:** 10.1097/MD.0000000000045702

**Published:** 2025-11-07

**Authors:** Na Liu, Shu Xu, Yuwen Xie, Ting Xu, Hehui Fang, Xiaoyue Wang, Liangfeng Yang, Shencun Fang

**Affiliations:** a Department of Respiratory Medicine, Nanjing Chest Hospital, The Affiliated Brain Hospital of Nanjing Medical University, Nanjing, China; b Department of Respiratory Medicine, Nanjing Gaochun People’s Hospital, Nanjing, China.

**Keywords:** aumolertinib, EGFR mutation, elderly, first-line therapy, NSCLC

## Abstract

Elderly non-small cell lung cancer (NSCLC) patients do not always benefit from standard treatments due to impaired organ function and/or multiple comorbidities. Our study aimed to determine the efficacy and safety of aumolertinib as a first-line therapy in NSCLC patients aged ≥65 and <65 years in clinical practice.We enrolled 100 patients with stage IIA–IVB epidermal growth factor receptor-mutant NSCLC who received aumolertinib alone as the first-line therapy. Efficacy and safety were compared between patients aged ≥65 and <65 years in different subgroups. The primary endpoint was the objective response rate (ORR). The secondary endpoints included progression-free survival (PFS), overall survival (OS), disease control rate (DCR), and safety.Overall, ORR and DCR were 76% and 98%, respectively. ORR was 69.4% and 82.4% for patients aged ≥ 65 and < 65 years, respectively (*P* = .27), and DCR was 98% and 98%, respectively (*P* = .93). The median PFS was 23.2 months. The median PFS was 26.9 months and 18.3 months in the ≥65 and <65 years groups, respectively (*P* = .377, and the median OS of all patients was 31.7 months. The median OS was 33.7 months and 30.7 months in the ≥65 and <65 years groups, respectively (*P* = .851). Adverse events were not statistically different between the 2 groups.The efficacy and safety profile of aumolertinib as a first-line therapy in elderly patients with epidermal growth factor receptor-mutant NSCLC were similar to those observed in the younger subgroup.

## 1. Introduction

Non-small cell lung cancer (NSCLC) is a malignant tumor with the highest incidence rate and mortality among people aged over 60 years in China and worldwide.^[1]^ Advanced NSCLC is the primary cause of death worldwide. Patients over 60 years of age account for more than 70% of newly diagnosed and deceased lung cancer patients across all age groups.^[2]^ Although novel and more effective therapies have improved the survival of lung cancer patients, as NSCLC is increasingly becoming a manageable chronic disease, the proportion of elderly patients within its population is consequently rising. However, elderly patients often have multiple comorbidities and decreased organ reserve function, which can have a significant impact on drug absorption, distribution, metabolism, and clearance, and may lead to reduced tolerance to treatment.^[3]^ Previous studies showed that the incidence and mortality rates of chemotherapy-induced granulocytopenia accompanied by infection are significantly higher in patients over 65 years of age.^[4]^ In addition, the risk and severity of chemotherapy-related adverse events (AEs) such as mucositis, cardiac toxicity, and neurotoxicity increase with age.^[5]^ Therefore, it is necessary to conduct more in-depth studies on elderly patients with NSCLC, especially on the efficacy and safety of first-line therapies. Currently, several epidermal growth factor receptor (EGFR)-tyrosine kinase inhibitors (TKIs) that have been approved by the Food and Drug Administration and National Medical Products Administration are widely used in treating patients with advanced NSCLC with EGFR-mutations. However, there is still controversy over whether EGFR-TKIs have the same effect on both elderly and younger patients.

Aumolertinib (HS-10296) is a novel, irreversible, third-generation EGFR-TKI developed by the Hansoh Pharmaceutical Group Co., Ltd (Shanghai, China). Aumolertinib can significantly inhibit both EGFR-sensitizing and T790M mutations and is also effective in patients with central nervous system (CNS) metastases. In the AENEAS (ClinicalTrials.gov identifier: NCT03849768) study, the median progression-free survival (PFS) was significantly longer in the aumolertinib group than in the gefitinib group,^[6]^ and the PFS benefit of aumolertinib was consistent across all predefined subgroups. Aumolertinib is a well-tolerated EGFR-TKI that can serve as a first-line therapy for EGFR-mutant NSCLC. Based on the AENEAS study, aumolertinib was approved in China as a first-line therapy for locally advanced or metastatic NSCLC with EGFR exon 19 deletions (19del) or L858R mutations in December 2021. However, current data on the use of third-generation EGFR-TKIs in elderly patients are limited. Consequently, the present study aimed to determine the efficacy and safety of aumolertinib as a first-line therapy in NSCLC patients aged ≥65 and <65 years in clinical practice.

## 2. Patients and methods

### 2.1. Patients

In this retrospective real-world study, we evaluated 100 EGFR-mutated NSCLC patients receiving aumolertinib alone as a first-line therapy at the Nanjing Chest Hospital between April 2020 and December 2023. The histological diagnosis and stage of NSCLC were based on the World Health Organization classification and tumor-node-metastasis (TNM) staging system,^[7]^ respectively. The performance status (PS) was estimated according to the Eastern Cooperative Oncology Group classification.

The pretreatment baseline evaluation included a complete medical history and physical examination, complete blood cell count, blood chemistry studies, thoracic and abdominal computed tomography, bone scintigraphy or 18F-fluorodeoxy-glucose positron emission tomography, and brain computed tomography or magnetic resonance imaging to evaluate the TNM stage. If patients were diagnosed with local NSCLC but were not suitable for thoracic surgery due to for example, poor PS or other diseases, we only included those treated with first-line aumolertinib in the present analysis. For all subjects fulfilling our inclusion criteria, an electronic clinical record search was performed at our hospital, and patient privacy was protected when using individual information. All patients were EGFR-TKI-naïve and received first-line aumolertinib (110 mg orally, once daily) until disease progression or intolerable toxicity. This study was performed in line with the principles of the Declaration of Helsinki. Approval was granted by the Ethics Committee of the Nanjing Chest Hospital.

### 2.2. Response evaluation and follow-ups

The Response Evaluation Criteria in Solid Tumors, version 1.1, was used for response assessment.^[8]^ PFS was defined as the period from the start of treatment until progressive disease or death occurred from any cause. Overall survival (OS) was defined as the time interval between the date of therapy initiation and the date of death from any cause or last follow-up. Adverse events associated with aumolertinib therapy were graded according to the Common Terminology Criteria for Adverse Events version 5.0.^[9]^ The primary endpoint was the objective response rate (ORR), while the secondary endpoints included disease control rate (DCR), PFS, OS, and safety.

### 2.3. Statistical analysis

Patients were stratified according to sex and disease stage, and efficacy and safety were compared between patients aged ≥65 and <65 years in different subgroups using the chi-square test. Survival curves were calculated using the Kaplan–Meier method. Statistical analyses were performed using SPSS version 11.0 for Windows. ORR and DCR were calculated at a 95% confidence level. The Cox proportional hazards model was used for univariate analysis of independent prognostic factors affecting PFS and OS, estimating the hazard ratio (HR) and its associated 95% CI. For the minimal missing data mentioned above, records with missing values for a specific variable were excluded from the analysis involving that variable. A *P* value of < 0.05 indicated statistical significance.

## 3. Results

### 3.1. Patient characteristics

Patient characteristics are summarized in Table 1. A total of 100 patients (42% males) were included in this study, and the median patient age was 66 years. Forty-nine patients were aged ≥65 years, and 51 patients were <65 years. Of all patients included, 68 had never previously smoked. According to the Eastern Cooperative Oncology Group criteria, 76 patients had a PS of 0 to 1, 14 had a PS of 2, and 10 had a PS of 3 to 4. The tumor type in 98 patients was adenocarcinoma. According to the TNM staging system, 6, 11, and 83 patients had stages II, III, and IV disease, respectively. In terms of EGFR-mutations, 53 and 43 patients had exon 19del and exon 21 L858R mutations, respectively.

**Table 1:** Patient characteristics (n = 100).

Characteristics	Number of patients
Age, yr	
≥65 yr	49 (49%)
<65 yr	51 (51%)
Gender	
Male	42 (42.0%)
Female	58 (58.0%)
Smoking status	
Never smoker	68 (68%)
Former/current smoker	29 (29%)
Unknown	3 (3%)
ECOG performance status	
0	10 (10%)
1	66 (66%)
2	14 (14%)
3	9 (9%)
4	1 (1%)
Histology	
Adenocarcinoma	98 (98%)
Squamous cell carcinoma	2 (2%)
Stage	
II	6 (6%)
III	11 (11%)
IV	83 (83%)
EGFR mutation	
Exon 19 deletion	53 (53%)
L858R	43 (43%)
Others	4 (4%)
Concurrent CNS metastases	
Yes	33 (33%)
No	67 (67%)

CNS = central nervous system, ECOG = Eastern Cooperative Oncology Group, EGFR = epidermal growth factor receptor.

### 3.2. Efficacy

Table 2 shows the response of EGFR-mutant NSCLC patients to first-line aumolertinib therapy. Three patients achieved complete response, while 73 patients met the criteria for partial response. Twenty-two patients showed stable disease during the observation period, and 2 patients showed progressive disease. Overall, ORR and DCR were 76.0% (95% CI: 66.4%–84.0%) and 98.0% (95% CI: 93.0%–99.8%), respectively, and were similar in both groups, with ORR of 69.4% (95% CI: 54.6%–81.7%) and 82.4% (95% CI: 69.1%–91.6%), respectively (*P* = .27), and DCR of 98.0% (95% CI: 89.1%–99.9%) and 98.0% (95% CI: 89.6%–100.0%), respectively (*P *= .93). In the subgroups with and without CNS metastases, the ORR was 69.7% (95% CI: 51.3%–84.4%) and 79.1% (95% CI: 67.4%–88.1%), respectively (*P* = .30).The DCR was 97.0% (95%CI: 84.2%–99.9%) and 98.5% (95% CI: 92.0%–100.0%), respectively (*P* = 1.00).

**Table 2:** Response to first-line aumolertinib therapy in EGFR-mutant NSCLC patients.

Response	Number of patients	Response rate (%, 95% CI)
Complete response	3	3
Partial response	73	73
Stable disease*	22	22
Progressive disease	2	2
Objective response rate		76.0 (66.4–84.0)
Disease control rate		98.0 (93.0–99.8)

* Stable disease was confirmed and sustained for 8 weeks or longer.

EGFR = epidermal growth factor receptor, NSCLC = non-small cell lung cancer.

**Figure 1.**
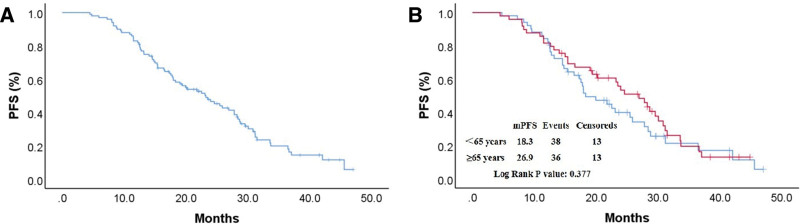
Kaplan–Meier estimates of PFS. (A) mPFS of all patients; (B) mPFS in the ≥65 and <65 years groups. PFS = median progression-free survival.

**Figure 2.**
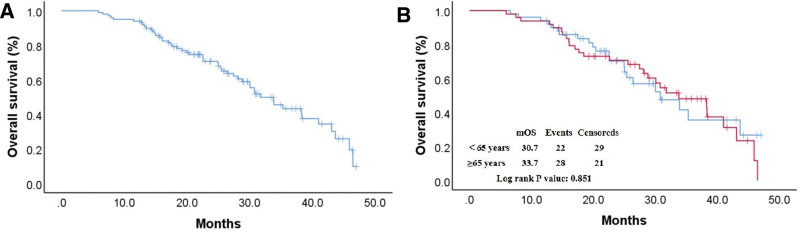
Kaplan–Meier estimates of OS. (A) mOS of all patients; (B) mOS in the ≥65 and <65 years groups. mOS = median OS, OS = overall survival.

The median follow-up time was 20.4 months (range 1.0–43.5 months). The PFS curves are shown in Figure 1A and B, and the median PFS (mPFS) of all patients was 23.2 months. The mPFS was 26.9 months and 18.3 months in the ≥65 and <65 years groups, respectively (*P* = .377). The HR between the 2 groups was 1.231 (95% CI, 0.776–1.593). The 2-year PFS rates were 44.9% and 29.4% in the 2 groups, respectively (*P *= .109). The OS curves are shown in Figure 2A and B, and the median OS of all patients was 31.7 months. The median OS was 33.7 months and 30.7 months in the ≥65 and <65 years groups, respectively (*P* = .851). The HR between the 2 groups was 0.948 (95% CI, 0.540–1.664). The 2-year OS rates were 59.2% and 43.1% in the 2 groups, respectively (*P* = .109). The presence or absence of CNS metastases had no significant impact on PFS (*P* = .106) or OS (*P* = .191).

Clinical factors related to prognosis were included in the Cox analysis. In the univariate analysis of intracranial progression-free survival, clinical stage was significantly associated with better PFS. Other factors, including age, were not significantly associated with PFS or OS (Tables S1–S2, Supplemental Digital Content, https://links.lww.com/MD/Q562).

### 3.3. Safety and AEs

Of the 100 patients included in the present study, 73 (73.0%) experienced at least 1 AE. A total of 40 patients (81.6%) in the ≥65 years group and 33 patients (64.7%) in the <65 years group experienced at least 1 treatment-emergent adverse event (TEAE). The most common TEAEs in the ≥65 and <65 years groups were increased blood creatine phosphokinase levels (24.5% vs 19.6%), rash (18.4% vs 15.7%), increased transaminase (20.4% vs 11.8%), and pruritus (16.3% vs 15.7%). Most AEs were mild. The incidence of grade ≥3 AEs was 5% (6/100), including 2 patients with elevated creatine phosphokinase, 1 with decreased platelet count, 1 with elevated transaminase, and 1 with chest tightness. All AEs were not significantly different between the 2 groups (*P* > .05) (Table 3).

**Table 3:** Summary of TEAEs (>5% of patients).

	TEAEs (any grade)		
	≥65 years (n = 49) No. (%)	<65 years (n = 51) No. (%)	Total (n = 100) No. (%)
CPK increase	12 (24.5%)	10 (19.6%)	22 (22%)
Rash	9 (18.4%)	8 (15.7%)	17 (17%)
Increased transaminase	10 (20.4%)	6 (11.8%)	16 (16%)
Pruritus	8 (16.3%)	8 (15.7%)	16 (16%)
Diarrhea	5 (10.2%)	4 (7.8%)	9 (9%)
Stomatitis	4 (8.2%)	3 (5.9%)	7 (7%)

CPK=creatine phosphokinase, TEAE = treatment-emergent adverse event.

## 4. Discussion

This study is the first to investigate the efficacy and safety of first-line aumolertinib in EGFR-mutant NSCLC patients across different age groups using real-world data. The results demonstrated no significant differences in PFS, OS, ORR, or DCR between patients aged ≥65 years and those <65 years, with a comparable safety profile, suggesting that age is not a significant factor influencing the efficacy or tolerability of aumolertinib. Subgroup analysis indicated that aumolertinib showed consistent efficacy regardless of the presence of CNS metastases at baseline, aligning with its favorable blood–brain barrier penetration capability. The mPFS of elderly patients in this study (23.2 months) was similar to that reported in the aumolertinib group in the randomized phase III AENEAS study (19.3 months).^[6]^ In addition, the benefits of aumolertinib use were consistent across all prespecified stratification factors, including patients ≥65 years old (HR, 0.54; 95% CI, 0.34–0.88) and <65 years old (HR, 0.44; 95% CI, 0.33–0.59) in the AENEAS study.

For elderly EGFR-mutant NSCLC patients, previous studies demonstrated that targeted therapy could improve prognosis, quality of life, and survival. In the combined analysis of North-East Japan Study Group studies, for patients aged ≥70 years, mPFS (14.3 vs 5.7 months, *P* < .001) and ORR (73.2% vs 26.5%, *P* < .001) were superior in the gefitinib group than in the standard chemotherapy group.^[10]^ Two additional studies, namely the OPTIMAL study^[11]^ and the EURTAC study,^[12]^ showed that elderly patients (≥65 years old) could benefit from erlotinib treatment compared with chemotherapy. More specifically, subgroup analyses of PFS revealed that HR was 0.17 and 0.28, respectively, in the 2 studies. However, erlotinib treatment for elderly patients (≥70 years old) had a higher incidence of dose discontinuation than younger patients in the BR.21 study due to AEs and grades 3 to 4 TRAEs.^[13]^

In contrast, second-generation EGFR-TKIs have shown limited benefits for elderly patients. In subgroup analyses of LUX-Lung 3, LUX-Lung 6, and LUX-Lung 7 trials, the afatinib group (patients ≥75 years old) did not exhibit any significant PFS (HR 0.69, 95% CI 0.331.44) and OS (HR 1.05, 95% CI 0.50–2.21) benefits compared with the gefitinib group. Moreover, the incidence of dose reduction (53% vs 5%) and treatment-related serious AEs (32% vs 14%) in the afatinib group was higher than that in the gefitinib group in patients aged ≥75 years old.^[14]^ In Asian patients in the ARCHER 1050 trial,^[15]^ PFS was significantly improved in the dacomitinib group compared with the gefitinib group in patients ≥65 years old (HR 0.598, 95% CI: 0.375–0.952); however, no significant difference in OS was noted (HR 1.051, 95% CI: 0.666–1.658). To date, there are no published safety data on dacomitinib use in patients aged ≥65 years.

Previous studies revealed significant differences in the efficacy and safety of third-generation EGFR-TKIs as a first-line therapy for elderly patients. Compared with the first-generation EGFR-TKI, the FLAURA study concluded that PFS was significantly improved in the osimertinib group in patients ≥65 years (HR 0.49, 95% CI: 0.35–0.67),^[16]^ but there was no significant difference in OS (HR 0.87, 95% CI: 0.63–1.22).^[17]^ Similarly, in the FURLONG study, furmonertinib showed no significant PFS benefit compared with gefitinib in patients ≥65 years old (HR 0.68, 95% CI: 0.43–1.09).^[18]^ Compared to icotinib, a randomized phase III study revealed that befotertinib did not significantly improve PFS in elderly patients (HR 0.79; 95% CI: 0.43–1.42).^[19]^ In contrast, aumolertinib showed remarkable efficacy in patients aged ≥65 years in both retrospective real-world studies and prospective randomized controlled studies. In this study, although the differences in median PFS (26.9 vs 18.3 months) and OS (33.7 vs 30.7 months) between the ≥65 and <65 age groups did not reach statistical significance, observable numerical differences were noted. These encouraging results clearly suggest that aumolertinib can be an efficient treatment option for a large number of elderly patients.

Targeted therapy has relatively fewer AEs and improved tolerance, rendering it the preferred systemic treatment for elderly patients with advanced NSCLC and driver gene mutations. In this study, toxicities associated with first-line aumolertinib therapy in elderly patients were generally mild and predictable. The incidence and severity were identical to those observed in patients aged <65 years. Although only 2 patients discontinued therapy due to TEAEs, several patients in the present study had other medical complications. Furthermore, the incidence of PS ≥ 2 was 24% in the present study. These patients had a relatively low tolerance to standard first-line chemotherapy. For frail patients (e.g., elderly patients with poor PS) with EGFR-mutated NSCLC, aumolertinib was a good alternative first-line therapy.

Based on these data, aumolertinib treatment for elderly patients with EGFR-mutations appears to be equally effective to that noted in younger populations. In clinical practice, aumolertinib can be actively considered as a first-line therapy for elderly patients with advanced NSCLC harboring EGFR-mutations. Individualized assessment and treatment planning should be conducted based on the patient’s specific circumstances. Additionally, it is crucial to strengthen our focus on elderly patients and provide them with improved treatment options.

This study has several limitations. Its retrospective design and small sample size may introduce potential biases. Additionally, the single-center nature of the study could limit the generalizability of the findings. Large-scale, multicenter, prospective studies are warranted in the future to validate the findings of this study.

## 5. Conclusion

In conclusion, our results demonstrate that first-line aumolertinib treatment shows promising efficacy and has acceptable toxicity in both elderly and younger patients with advanced NSCLC harboring EGFR mutation. Although this was a retrospective real-world study, our results suggest that first-line aumolertinib should be considered as a preferable standard treatment in elderly patients.

## Author contributions

**Conceptualization:** Na Liu, Shu Xu, Shencun Fang.

**Data curation:** Yuwen Xie, Ting Xu.

**Formal analysis:** Na Liu, Hehui Fang, Xiaoyue Wang.

**Investigation:** Shu Xu, Liangfeng Yang.

**Supervision:** Shencun Fang.

**Writing – original draft:** Na Liu, Shu Xu, Shencun Fang.

**Writing – review & editing:** Na Liu, Shu Xu, Yuwen Xie, Ting Xu, Shencun Fang.

## Supplementary Material


