# Supplementary material for: Real-world study of first-line therapy with aumolertinib for elderly patients with non-small cell lung cancer harboring EGFR mutation
Source: Medicine (Baltimore). 2025 Nov 7;104(45):e45702. doi: 10.1097/MD.0000000000045702 (PMC12599707; doi:10.1097/MD.0000000000045702)
Supplement: Supplementary file 1 [file medi-104-e45702-s001.docx]

**Supplementary Table 1** Prognostic factors for PFS

| Variables |  |  | Univariate |  |  |  | Multivariate |  |  |
| --- | --- | --- | --- | --- | --- | --- | --- | --- | --- |
|  |  |  | HR(95%CI) |  | P |  | HR(95%CI) |  | P |
| Age(<65 vs. ≥65) | | | 0.782(0.493-1.239) | | 0.295 |  |  |  |  |
| Gender (male vs. female) | | | 0.850(0.536-1.348) | | 0.491 |  |  |  |  |
| EGFR mutation(19del vs. | | | 1.245(0.774-2.003) | | 0.366 |  |  |  |  |
| L858R) | | |  | |  |  |  |  |  |
| Smoking (yes vs.no) | | | 1.249(0.764-2.042) | | 0.375 |  |  |  |  |
| Stage(II-III vs.IV) | | | 2.423(1.193-4.921) | | **0.014** |  |  |  |  |
| ECOG PS (<2vs.≥2） | | | 1.297(0.773-2.175) | | 0.324 |  |  | |  |
| Concurrent CNS metastases | | | 1.491(0.915-2.430) | | 0.109 |  |  |  |  |

**Supplementary Table 2** Prognostic factors for OS

| Variables |  |  | Univariate |  |  |  | Multivariate |  |  |
| --- | --- | --- | --- | --- | --- | --- | --- | --- | --- |
|  |  |  | HR(95%CI) |  | P |  | HR(95%CI) |  | P |
| Age(<65 vs. ≥65) | | | 1.061(0.603-1.866) | | 0.838 |  |  |  |  |
| Gender (male vs. female) | | | 0.783(0.447-1.373) | | 0.394 |  |  |  |  |
| EGFR mutation(19del vs. | | | 1.496(0.834-2.684) | | 0.177 |  |  |  |  |
| L858R) | | |  | |  |  |  |  |  |
| Smoking (yes vs.no) | | | 0.765(0.423-1.384) | | 0.376 |  |  |  |  |
| Stage(II-III vs.IV) | | | 1.470(0.685-3.154) | | 0.323 |  |  |  |  |
| ECOG PS (<2vs.≥2） | | | 1.380(0.751-2.535) | | 0.300 |  |  | |  |
| Concurrent CNS metastases | | | 1.484(0.817-2.696) | | 0.195 |  |  |  |  |
